# A Simplified Streptozotocin-Induced Diabetes Model in Nude Mice

**DOI:** 10.32607/actanaturae.11202

**Published:** 2020

**Authors:** I. G. Gvazava, A. V. Kosykh, O. S. Rogovaya, O. P. Popova, K. A. Sobyanin, A. C. Khrushchev, A. V. Timofeev, E. A. Vorotelyak

**Affiliations:** Koltsov Institute of Developmental Biology, Russian Academy of Sciences, Moscow, 119334 Russia; National Medical Research Treatment and Rehabilitation Centre, Ministry of Health of the Russian Federation, Moscow, 125367 Russia; Pirogov Russian National Research Medical University, Ministry of Health of the Russian Federation, Moscow, 117997 Russia

**Keywords:** animal model, Nude mice, diabetes mellitus, streptozotocin

## Abstract

Preclinical studies of human cellular and tissue-based products (HCT/Ps) for transplantation therapy of type 1 diabetes mellitus (T1DM) necessarily involve animal models, particularly mouse models of diabetes induced by streptozotocin (STZ). These models should mimic the clinical and metabolic manifestations of T1DM in humans (face validity) and be similar to T1DM in terms of the pathogenetic mechanism (construct validity). Furthermore, since HCT/Ps contain human cells, modeling of diabetes in immune-deficient animals is obligatory. Here we describe the most simplified diabetes model in Nude mice. Diabetes was induced in 31 males by a single intraperitoneal injection of STZ in normal saline at a medium-to-high dose of 150 mg/kg body weight. Fourteen control animals received only saline. Non-fasting plasma glucose (PG) levels were measured periodically for 50 days. All STZ-treated mice survived beyond 50 days. By day 15 after STZ administration, 22 of 31 (71%) mice developed stable diabetes based on the following criteria: (1) non-fasting PG ≥ 15 mmol/L on consecutive measurements up until day 50; (2) no diabetes remission. The mean non-fasting PG in mice with stable diabetes over the period of 35 days was equal to 25.7 mmol/L. On day 50, mean plasma insulin concentration, mean pancreatic insulin content, and the average number of β-cells in pancreatic islets were 2.6, 8.4, and 50 times lower, respectively, than in the control animals. We consider that our Nude mouse model of diabetes meets face validity and construct validity criteria and can be used in preclinical studies of HCT/Ps.

## INTRODUCTION

Over the past two decades, considerable progress has been made in the development of human cellular and tissue-based products (HCT/Ps) for the transplantation therapy of type 1 diabetes mellitus (T1DM) [1]. Preclinical studies of these HCT/Ps require the assessment of their antidiabetic (glucose-lowering) effect in animal models of diabetes. Streptozotocin (STZ)-induced diabetic mouse models are the ones used most commonly. This is due to their simplicity, low cost, and, most importantly, their pathogenetic and phenotypic adequacy [2, 3]. Pathogenetic adequacy implies similarity between the developmental mechanisms of STZ-induced diabetes in mice and T1DM in humans. In both cases, the disease is caused by the destruction of β-cells, resulting in insulin deficiency. Phenotypic adequacy refers to the similarity between the manifestations of STZ-induced diabetes and type 1 diabetes: mice develop hyperglycemia; the number of β-cells in the islets of Langerhans decreases sharply; polyuria, polydipsia, weight loss, and decreased viability are observed.

There are two main methods for diabetes induction by streptozotocin in mice: repeated low-dose administration of streptozotocin (40–60 mg/kg of animal weight) for 4–5 days and a single administration of a medium to high dose (100–250 mg/kg). The first method is slightly more efficient, though more laborious [2]. STZ is injected intraperitoneally or intravenously via either one of the tail veins or the penile vein (for males). For the intraperitoneal injection, there is a risk of accidentally injuring the intestine, which leads to animal death. At the same time, possible penetration of STZ into the subcutaneous tissue rather than the peritoneal cavity weakens the diabetogenic effect of STZ [4]. Nevertheless, intraperitoneal administration of STZ is used much more often than intravenous injection, as the former method is simpler.

Being structurally and conformationally similar to glucose, STZ enters murine β-cells via the glucose transporter GLUT2. Since STZ competes with glucose for the uptake by this transporter, it is recommended not to feed the animals for at least 4 h prior to STZ administration in order to increase the efficiency of diabetes induction [5]. However, Chaudhry et al. showed that the effectiveness of diabetes induction by STZ is the same in both fed and fasting C57BL/6 and NOD/ SCID mice [6]. Administration of STZ to fed mice is preferable, since it allows one to eliminate the stress caused by starvation.

STZ is believed to rapidly lose its activity in neutral pH solutions. For this reason, many protocols recommend dissolving STZ in citrate buffer with a pH of 4–4.5 to induce diabetes [5, 7]. Even a small volume of citrate buffer at such low pH can cause peritoneal irritation and significantly shift the acid-base equilibrium. Therefore, many researchers use pH-neutral media (phosphate-buffered saline, Hanks’ balanced salt solution, and 0.9% NaCl) to dissolve STZ [4, 6, 8].

It is important to note that mice challenged with high-dose STZ (> 200 mg/kg) rapidly develop dehydration (due to hyperglycemia and the general toxic effect of STZ) and severe hypoglycemia (caused by a massive release of insulin from destroyed β-cells). Subcutaneous injections of saline solutions are used to correct the water-electrolyte imbalance; a sucrose solution is administered orally to eliminate hypoglycemia [5, 9]. These measures are not necessary when using lower-dose STZ.

Studying the antidiabetic effect of HCT/Ps in diabetic mice involves a number of challenges:

•manifestation of the HCT/P effect usually requires quite a long time, from several weeks to several months. During this period, mice should maintain stable diabetes; i.e., the rate of spontaneous remission of the disease should be as low as possible;

•blood glucose levels in diabetic mice should be much higher than those in intact animals: it is the only way to confidently determine the effect of HCT/Ps;

•in order to study the effects of different doses of HCT/Ps and/or different methods of their transplantation, it is obligatory to have many groups of animals with stable diabetes while the size of each group should ensure the statistical reliability of the results. Therefore, the effectiveness of diabetes induction (morbidity) should be maximized, while the diabetes mortality rate should be minimized;

•increasing the STZ dose to enhance the effectiveness of diabetes induction raises the mortality rate among mice. Mortality can be reduced by constant therapy with low-dose insulin administration [9, 10]; however, this complicates the handling of the animals and makes it difficult to assess the effects of HCT/Ps;

•any HCT/P contains human cells, which are xenogeneic to recipient mice. For this reason, animals resistant to xenoantigens (and Nude mice in particular) are used to study antidiabetic HCT/Ps. The data on the suitability of Nude mice for modeling diabetes with STZ are rather controversial. Some researchers consider that these mice are especially vulnerable to the toxic effect of STZ because of their genetic aberrations [7]. Others believe that Nude mice are quite convenient for diabetes modeling with streptozotocin but still use insulin therapy to improve animal survival [9].

The aim of our study was to find the simplest and most reliable Nude mouse model of diabetes. The main problem needing a solution before any work could start was choosing the proper STZ dose. An analysis of the published data showed that stable diabetes can be induced in Nude mice from different breeders by a single administration of STZ at a dose range of 160–240 mg/kg. However, high animal mortality was observed when using such doses; it ranged from 7% to 100% for a period of 30 days after STZ injection [4, 9, 11, 12]. For this reason, we decided to use a lower dose of STZ. We conducted preliminary experiments in C57BL/6 mice and found that STZ at a dose of 150 mg/kg provides an acceptable incidence of diabetes and almost a 100% survival rate (unpublished data). This was the dose used to induce diabetes in Nude mice in the present study.

## EXPERIMENTAL

**Animals**

Male Nude Crl:NU(NCr)-*Foxn1nu *mice (age, 15–18 weeks; average weight, 31.5 ± 3.3 g) purchased from Charles River Breeding Laboratories (Germany) were used. All work with mice was performed under SPF conditions. The animals received sterilized chaw and water *ad libitum*. Mice were maintained at a temperature of 20–25°C on a 12:12 h light/dark cycle. All the experiments were carried out in accordance with the Guide for the Care and Use of Laboratory Animals of Pirogov Russian National Research Medical University dated March 27, 2019, in compliance with European Directive 2010/63/EU on the protection of experimental animals.

**Method of diabetes induction**

The animals were divided into two groups: the experimental (D, *n *= 31) and control ones (C, *n *= 14). In group D mice, diabetes was induced by a single intraperitoneal injection of STZ (Sigma S0130, USA) at a dose of 150 mg/kg; mice were deprived of food 4 h prior to administration. STZ was dissolved in cold 0.9% NaCl immediately before the injection; the injection volume was 450–550 μL. Group C mice were injected with 0.9% NaCl.

**Methods for assessing the diabetogenic effect of STZ**

In all animals, non-fasting PG was determined prior to STZ administration (on day 0), as well as on days 8, 10, and then every 5 days until day 50 after STZ administration in the time period between 13:00 and 15:00. A Contour TS glucose meter and corresponding test strips (Bayer, Switzerland) were used to measure PG. The diagnostic performance of the glucose meter and test strips was assessed periodically using control Contour solutions with low, normal, and high glucose concentrations. Blood samples for PG measurements were taken from tail tips. The High symbol was displayed on the screen at PG > 33.3 mmol/L. In such cases, the PG was considered equal to 33.3 mmol/L.

Diabetes was diagnosed when PG was equal to or exceeded 15 mmol/L for two consecutive readings (e.g., on days 8 and 10). Diabetes was considered stable if PG ≥ 15 mmol/L was obtained in all measurements between days 15 and 50. Diabetes remission was established if PG was below 15 mmol/L in at least one measurement on days 40 through 50.

On day 50, the intraperitoneal glucose tolerance test (IPGTT) was performed in group D mice with stable diabetes and in group C mice. Glucose dissolved in 500 μL of 0.9% NaCl was injected at a dose of 2 g/kg. At minutes 0 (prior to glucose injection), 15, and 60 of the test, mice were anesthetized with isoflurane (Baxter Healthcare Corporation, USA). Next, thoracotomy was performed, and 200–00 μL of blood was collected from the heart chambers into a lithium heparin tube (Microvette 500-LH, Sarstedt, Germany) using a 25G needle. PG was measured in the whole blood. The sample was then centrifuged, and the plasma insulin level was measured by ELISA (Mercodia, Sweden). After blood sampling, the mice were sacrificed by cervical dislocation.

Simultaneously with blood sampling at minute 0 of IPGTT, the pancreas was removed from the sacrificed mice and divided into three fragments. The first fragment was fixed in 10% neutral formalin (BioVitrum, Russia), embedded in paraffin, and then cut into 4- to 5-•µm-thick sections. The sections were incubated with mouse anti-insulin antibodies (1 : 1000; catalog # 035K4884, Merck/Sigma, USA). Insulin-positive cells were detected using an EnVision FLEX kit (Agilent/ Dako K8000, Denmark). The second fragment was frozen in liquid nitrogen, and 4-μm cryostat sections were prepared. These sections were sequentially incubated with rabbit anti-insulin antibodies (1 : 200; catalog # ab181547, Abcam, UK) and anti-rabbit Ig antibodies (1 : 500; Invitrogen Alexa Fluor Plus 488, A32790; ThermoFisher Scientific, USA). Next, the sections were mounted in Vectashield Antifade Mounting Medium with the DAPI fluorescent dye (H-1200, Vector Laboratories, USA). Immunomorphological studies were performed using a Nikon Eclipse 80i microscope (Nikon, Japan). The third fragment of the pancreas was used to assess the insulin content in the pancreatic tissue. The fragment was dried, weighed, minced with scissors in a minimal volume of water, and then sonicated. Insulin was extracted from the resulting suspension with a mixture of ethanol and hydrochloric acid [13]. Insulin concentration in the extract was measured by ELISA and normalized to the weight of the fragment.

The weight of the mice was measured in all groups at the beginning and end of the observation period.

**Methods of statistical data processing and analysis**

We used the MedCalc Statistical Software (version 19.4.0, MedCalc Software Ltd, Ostend, Belgium; https://www.medcalc.org; 2020). The normal distribution of data was assessed using the Shapiro–Wilk test. Intergroup differences were analyzed using the two-tailed Student’s *t*-test in the case of a normal distribution of data and homogeneity of variance. Welch’s* t*-test was used in case of a normal data distribution and heterogeneity of variance. In all cases, the level of significance of the differences was considered equal to 5% (α error = 0.05). The Kaplan–eier plot analysis was used to estimate the diabetes incidence. The results of our measurements of PG, animal weight, plasma insulin levels, and insulin content in the pancreas are presented as a mean ± standard deviation with 95% confidence intervals for the means in the text and as mean ± standard deviations in figures.

## RESULTS AND DISCUSSION

**Effectiveness of diabetes induction**

**Fig. 1:**
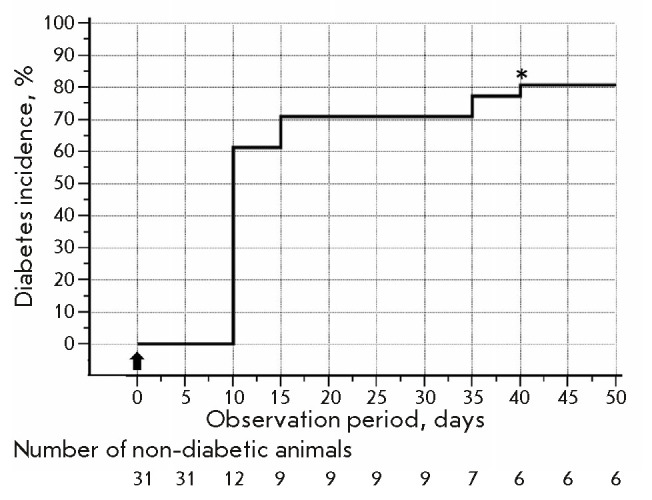
Diabetes incidence in group D (Kaplan–Meyer analysis). The arrow indicates STZ injection; the asterisk marks the onset of diabetes remission in one of the animals

During the entire observation period, diabetes developed in 25 mice in group D (*Fig. 1*). However, stable diabetes was noted in only 22 animals. Thus, the effectiveness of induction of stable diabetes amounted to 71%. One mouse with late onset of diabetes developed remission; no remission was observed in mice with stable diabetes. The median incidence was 10 (10–15) days. None of the group D animals died within 50 days after STZ administration.

It is difficult to compare our data on the effectiveness of diabetes induction and survival rate to the results of other studies, since Nude mice from other breeders and administered different doses of STZ were used in these studies. For instance, Deeds et al. [4] conducted experiments in mice obtained from Taconic Farms (USA). After having received an STZ dose of 220 mg/kg, 92.5% of the animals developed severe diabetes on day 5; however, the mortality rate by day 20 was 20%. In a study by Graham et al. [9], Charles River mice (USA) developed stable diabetes on day 5 after administration of 240 mg/kg of STZ, while the mortality rate by day 30 was as low as 8%. However, such a low mortality rate might be explained by the fact that the animals received insulin therapy during the study period. In the study by Zhao et al. [12], the effectiveness of diabetes induction in mice purchased from the Shanghai Slacass breeding nursery (China) was 100% on day 8 after injection of 200 mg/kg of STZ; however, all mice died on day 30. Thus, our medium-dose model of diabetes is inferior to high-dose models in terms of the effectiveness of disease induction but superior to them in such an important parameter as animal survival.

**Changes in PG**

**Fig. 2:**
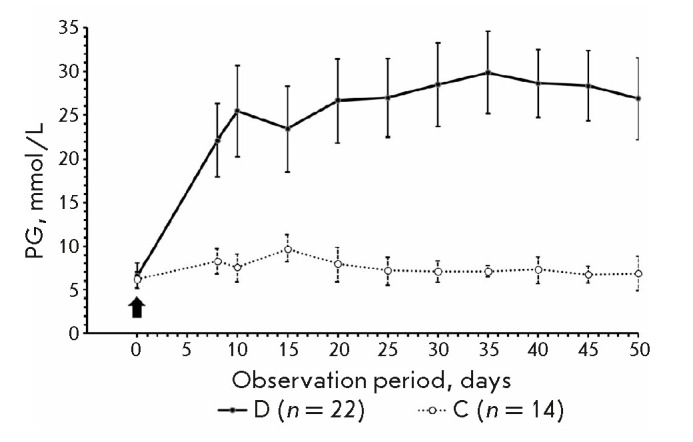
Non-fasting PG values in group D mice with stable diabetes and group C mice during the observation period. The arrow indicates STZ injection

Hyperglycemia in the diabetic range (PG ≥ 15 mmol/L) was observed in group D mice with stable diabetes starting from day 8 after STZ administration (*Fig. 2*). The mean group levels of PG for the entire observation period in group D mice with stable diabetes and in group C mice were 25.7 Ѓ} 3.5 (24.1–27.2) mmol/L and 7.5 ± 0.3 (7.1–7.8) mmol/L, respectively. The areas under the PG curves for the entire observation period were 1,258 ± 172 (1,184–1,332) and 365 ± 13 (349–382) mmol/L × 50 days, respectively (*P* < 0.0001 in both cases; Student’s *t*-test). Our results of PG evaluation in the groups D and C are similar to those obtained by Deeds et al. [4]. In this study, the mean baseline PG in fed Nude mice was 7.7 Ѓ} 1.1 mmol/L. It increased to 28.6 Ѓ} 5.3 mmol/L seven days after STZ administration and remained at this level for 20 days.

**Weight changes in mice**

**Fig. 3:**
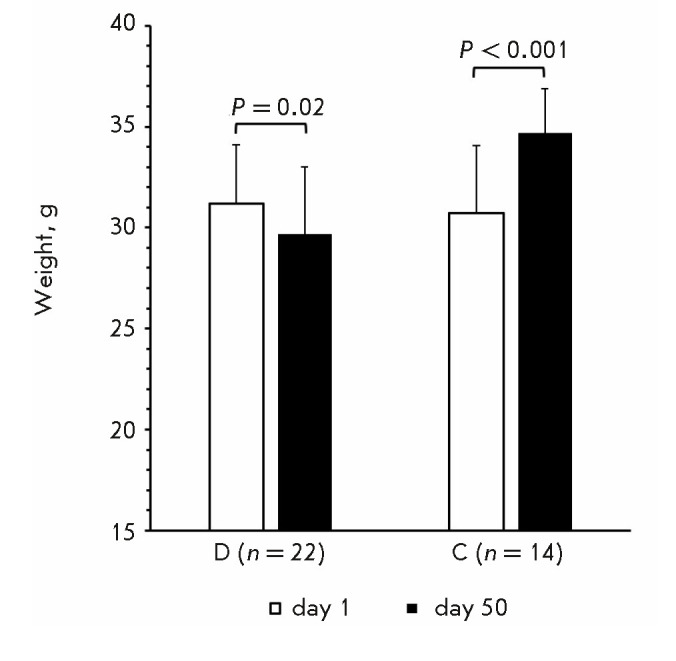
Body weight of group D mice with stable diabetes and group C mice at the beginning and at the end of the observation period

By the end of the observation period, the weight of mice with stable diabetes had decreased by an average of 4.8 ± 0.9%, while the weight of group C mice increased by 13 ± 5.8% (*Fig. 3*). Weight loss in STZ-induced diabetic rodents has been well documented and needs no further discussion.

**The results of the intraperitoneal glucose tolerance test (IPGTT)**

**Fig. 4:**
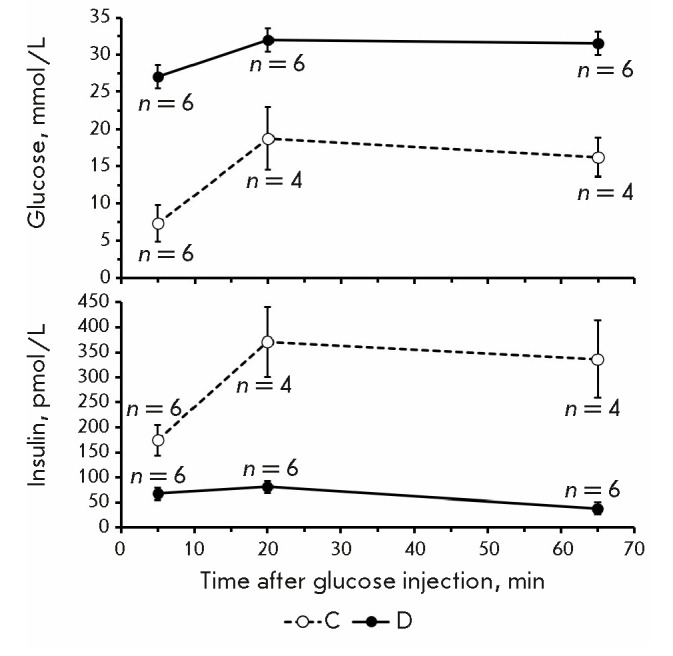
Changes in PG and plasma insulin content during IPGTT in group D mice with stable diabetes and group C mice

The basal insulin levels (at minute 0 of IPGTT) in group D mice with stable diabetes were 2.6 times lower than those in group C mice, equal to 67 ± 17 (49–85) pmol/L and 174 ± 31 (141–207) pmol/L, respectively (*P * < 0.0001; Student’s *t*-test) (*Fig. 4*).

The areas under the PG curves in mice with stable diabetes and intact mice were 1,870 ± 108 (1,757–1,982) and 996 ± 160 (827–1,163) mmol/L × 60 min, respectively; the areas under the insulin level curves were 3,770 ± 849 (2,879–4,661) and 20,008 ± 4,052 (15,755– 24,260) pmol/L × min, respectively (*P* < 0.0001 in both cases; Student’s *t*-test).

The changes in PG and insulin levels observed by us during IPGTT in intact Nude mice were close to those in similar tests carried out both in Nude mice and mice of other strains. Thus, in a study by Christoffersson et al. [14], the highest PG in intact Nude mice recorded 15 min after the intraperitoneal glucose injection at a dose of 2.5 mg/kg was approximately 17 mmol/L, while the area under the PG curve was approximately 800 mmol/L × 60 min. Harper et al. [15] showed that insulin levels in intact outbred mice obtained from different breeders at minute 0 varied between 120 and 200 pmol/L, and maximum insulin levels were attained at minute 15 after glucose administration and ranged from 165 pmol/L to 280 pmol/L.

In our study, the plasma insulin levels in mice with stable diabetes were quite significant at all stages of IPGTT. Therefore, even in the presence of severe diabetes, Nude mice retain a certain number of functionally active β-cells. Residual insulin secretion is also observed in patients with type 1 diabetes for several years after clinical manifestation of the disease [16]. Thus, the presence of insulin in the plasma of group D mice with stable diabetes confirms the phenotypic similarity of our diabetic model to T1DM.

**Insulin content in the pancreas**

**Fig. 5:**
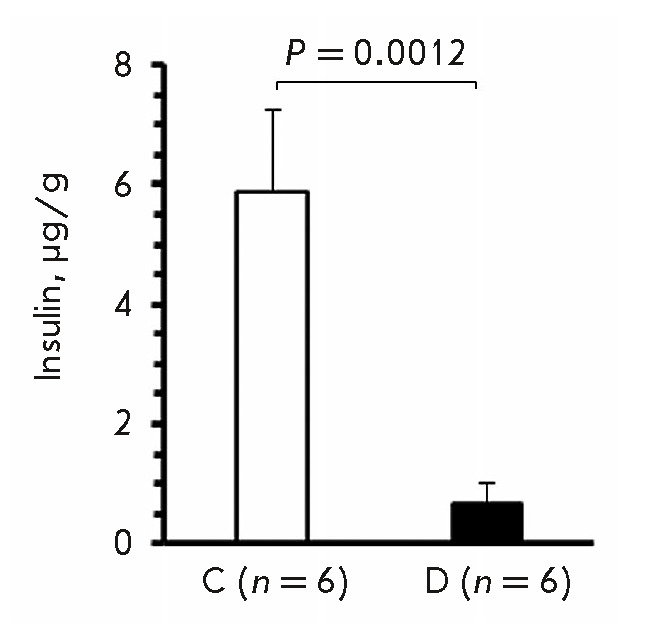
Insulin content in the pancreatic tissue of group D mice with stable diabetes and group C mice on observation day 50

On day 50, the mean insulin levels in the pancreas of group D mice with stable diabetes and group C mice were 0.7 ± 0.3 (0.2–1.1) and 5.9 ± 0.6 (4.2–7.7) μg/g of the gland weight, respectively; *P* < 0.0001; Welch’s* t*-test (*Fig. 5*).

According to the published data, the insulin content in the mouse pancreas varies widely: it ranges from 2.5 to 80 μg/g of pancreatic weight in healthy animals and from 0.2 to 20 μg/g of pancreatic weight in animals with STZ-induced diabetes [3, 8, 12]. This wide fluctuation is due to interlinear, age, and sex differences in animals, different duration and severity of the diabetes, as well as the variety of the samples (entire pancreas, individual pancreatic lobes) and methods used for insulin extraction. Ultimately, when assessing the degree

**Microscopic studies of the pancreas**

**Fig. 6:**
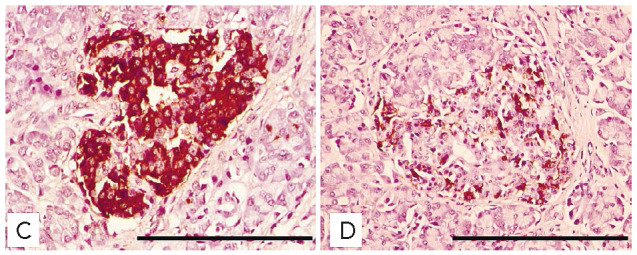
Pancreatic islets in group D mice with stable diabetes and group C mice on day 50. Light microscopy, immunohistochemical staining for insulin, 400× magnification. Scale bar, 100 μm

**Fig. 7:**
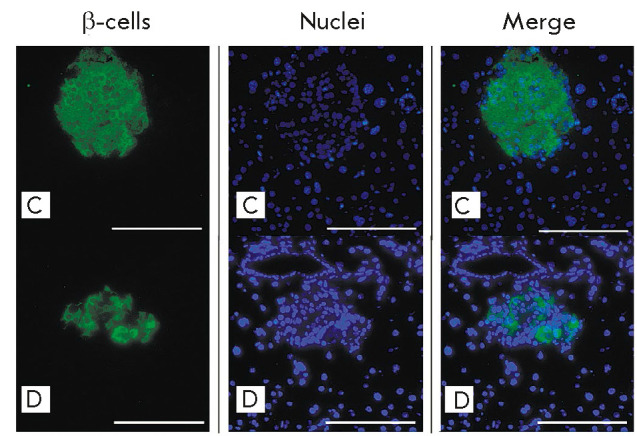
Pancreatic islets in group D mice with stable diabetes and group C mice on day 50. Fluorescent microscopy; β-cells were stained for insulin (green); nuclei were stained with DAPI (magenta); 200× magnification. Scale bar, 100 μm

By observation day 50, the number of islet β-cells had greatly decreased, and foci of intra- and peri-insular sclerosis occurred in animals with stable diabetes (*Fig. 6*). By having directly counted β-cells (*Fig. 7*), we found that their number in the islets of mice with stable diabetes had decreased about 50-fold compared to the control. A similar pathomorphological pattern is typical of diabetes induced by the administration of a single medium or high dose of STZ to mice [4, 12].

## CONCLUSIONS

The advantages of our diabetes model are as follows:

•the use of Nude mice allows for transplantation of xenogeneic HCT/Ps containing human cells in animals;

•the method of diabetes induction is simplified as much as possible: STZ is administered once intraperitoneally;

•since 0.9% NaCl is used instead of a low-pH buffer solution to dissolve STZ, the risk of peritoneal irritation is eliminated while the general toxic effect of STZ is reduced;

•the effectiveness of diabetes induction is approximately 71%, while the survival rate is 100%. This makes it possible to form several experimental groups of mice with a group size sufficient to obtain statistically reliable experimental data;

•the use of medium-to-high doses of STZ requires neither correction of the water-electrolyte balance nor maintenance of insulin therapy;

•stable diabetes persists for a long time: from day 15 to day 50 after STZ administration. This period is sufficient to assess the antidiabetic effect of HCT/Ps;

•PG values are measured in fed animals. This eliminates the stress caused by prolonged starvation to animals;

•in animals with stable diabetes, PG is much higher than that in the control animals and there is also no spontaneous remission of the disease, thus simplifying the assessment of the antidiabetic effect of HCT/Ps;

•the model is phenotypically and pathogenetically similar to T1DM in humans; and

•the model allows one to conduct the biochemical, hormonal, and pathomorphological studies required in order to assess the antidiabetic effect of HCT/Ps.

## Abbreviations

HCT/P: human cellular and tissue-based product
IPGTT: intraperitoneal glucose tolerance test
PG: plasma glucose level
STZ: streptozotocin
T1DM: type 1 diabetes mellitus
